# Non-Inferiority Trials: A Systematic Review on Methodological Quality and Reporting Standards

**DOI:** 10.1007/s11606-024-08890-9

**Published:** 2024-07-01

**Authors:** Anthony Sengul, Edison Escobar, John R. Flores, Michelle Kwok, Shogo Kono, Gordon Guyatt, Cynthia A. Jackevicius

**Affiliations:** 1 Department of Pharmacy Practice and Administration, Western University of Health Sciences, Pomona, CA USA; 2 Kaiser Permanente Santa Monica, Santa Monica, CA USA; 3 Kaiser Permanente San Bernardino County, Fontana, CA USA; 4 Walgreens, Chino Hills, CA USA; 5 VA Greater Los Angeles Healthcare System, Los Angeles, CA USA; 6 Department of Health Research Methods, Evidence, and Impact, McMaster University, Hamilton, ON Canada; 7 Institute for Health Policy, Management and Evaluation, University of Toronto, Toronto, Canada; 8 ICES, Toronto, Canada

**Keywords:** non-inferiority, methodological quality, reporting standards

## Abstract

**Background:**

Non-inferiority (NI) trials require unique trial design and methods, which pose challenges in their interpretation and applicability, risking introduction of inferior therapies in clinical practice. With the abundance of novel therapies, NI trials are increasing in publication. Prior studies found inadequate quality of reporting of NI studies, but were limited to certain specialties/journals, lacked NI margin evaluation, and did not examine temporal changes in quality. We conducted a systematic review without restriction to journal type, journal impact factor, disease state or intervention to evaluate the quality of NI trials, including a comprehensive risk of bias assessment and comparison of quality over time.

**Methodology:**

We searched PubMed and Cochrane Library databases for NI trials published in English in 2014 and 2019. They were assessed for: study design and NI margin characteristics, primary results, and risk of bias for blinding, concealment, analysis method and missing outcome data.

**Results:**

We included 823 studies. Between 2014 and 2019, a shift from publication in specialty to general journals (15% vs 28%, p < 0.001) and from pharmacological to non-pharmacological interventions (25% vs 38%, p = 0.025) was observed. The NI margin was specified in most trials for both years (94% vs 95%). Rationale for the NI margin increased (36% vs 57%, p < 0.001), but remained low, with clinical judgement the most common rationale (30% vs 23%), but more 2019 articles incorporating patient values (0.3% vs 21%, p < 0.001). Over 50% of studies were open-label for both years. Gold standard method of analyses (both per protocol + (modified) intention to treat) declined over time (43% vs 36%, p < 0.001).

**Discussion:**

The methodological quality and reporting of NI trials remains inadequate although improving in some areas. Improved methods for NI margin justification, blinding, and analysis method are warranted to facilitate clinical decision-making.

**Supplementary Information:**

The online version contains supplementary material available at 10.1007/s11606-024-08890-9.

## INTRODUCTION

In modern medicine, clinical trials are imperative in guiding clinical decision-making. However, differences exist in the design, analysis, and interpretability of such trials, for example, between superiority and noninferiority (NI) trials, as outlined in available guidelines.^1^ Despite prior studies highlighting concerns related to trial design and validity, NI trials are increasing in publication, particularly in areas where effective standards of therapy already exist, making placebo-controlled trials not ethically feasible.^2^ Further, many of these novel therapies are not expected to be better compared with the standard of care, limiting the utility of superiority trials. NI trials then aim to demonstrate that a treatment is not that much worse than an active control by more than a pre-specified amount known as the NI margin (NIM), a measure of loss of efficacy. To justify this potential loss of efficacy, it is essential that the novel therapy in a NI trial offers some other advantage compared with the standard of care, such as reduced cost, improved safety or greater convenience. As such, a NI trial must be conducted and interpreted properly, or it risks exposing patients to potentially inferior therapies.

However, NI trials can pose unique challenges as they require specific trial design, conduct, analysis and interpretation. The 2010 extended Consolidated Standards of Reporting Trials (CONSORT) guide the conduct and reporting of NI trials.^1,3^ However, previous studies have demonstrated lack of adherence to these guidelines which may lead to deficiencies in NI study design and quality of reporting, including lack of NI margin justification and unsuitable analysis methods.^4,5^ Such deficiencies can impact the accuracy, interpretability, and applicability of NI study results. Thus, it is imperative that these minimum standards are met to facilitate clinical decision-making.

Prior studies evaluating the methodological quality and reporting standards of published NI studies were limited to certain specialties or journal types, examined trials published before 2010, lacked evaluation of the NI margin, and did not examine temporal changes in quality over time.^4,6^ Thus, we conducted a systematic review to evaluate the methodological quality and reporting standards of more recently published NI trials without restriction to journal type or specialty, providing a broad-based evaluation of contemporary NI trials. Further, we examined trends in the methodological quality and reporting of NI trials over time.

## METHODS

### Study Design

We conducted a systematic review of non-inferiority randomized controlled trials using PRISMA methods.

### Study Eligibility

We included noninferiority, randomized controlled trials that were published in full-text in English in 2014 and 2019 without restriction to journal type or medical specialty. We excluded articles that only had an abstract or were equivalence or bioequivalence studies, design papers, meta-analyses, review articles, editorials, diagnostic trials, non-human trials, and duplicates.

### Search Strategy

We electronically searched PubMed and Cochrane Library databases to identify randomized controlled trials published in 2014 and 2019. We searched PubMed using these key words: (noninferiority, non-inferiority, noninferior, non-inferior, noninferior*, non-inferior*) AND random*, and applied filters for human studies and English language studies. We searched Cochrane Library using the advanced search strategy using these key words: (noninferiority* non-inferiority* noninferior* non-inferior*) AND random*, and applied search limits for content type (trials), Cochrane Library publication date (all dates) and CENTRAL Trials only original publication year (2014 or 2019) and identified EMBASE as our preferred source to limit our search.

### Article Selection

One of three investigators individually screened all titles and abstracts to identify potentially relevant studies. One of three investigators reviewed the remaining articles in full-text to make the final decision for article selection. Any uncertainties were reviewed by a fourth investigator and resolved by consensus.

### Data Abstraction

Data were abstracted by three independent reviewers using a standardized data extraction form. Reviewers were first trained on a common set of 3 articles, iteratively, until an agreement rate for abstraction was at least 90%. After each iteration, the reviewers discussed interpretation of the items where differences existed. Revisions to the data abstraction dictionary were made at each iteration to ensure a common understanding of data abstraction. Reviewers were not blinded to journal name and author names. Any disagreement was discussed with a fourth study investigator, and differences were resolved through discussion and by consensus.

Data were collected directly from published NI studies and their associated design papers, protocols, and appendices (if available). Data abstracted included: 1) characteristics of the trial (medical focus [general medicine vs specialty], year of publication, journal, journal impact factor, sample size, intervention [using the American Hospital Formulary Service (AHFS) classification of pharmacological therapies], control, primary outcome studied, type of outcome [surrogate, such as, blood pressure; clinical, such as, stroke]); 2) study design characteristics to assess compliance with the NI CONSORT statement (risk of bias for study design criteria [randomization, concealment, blinding, missing outcome data], analysis method [intention to treat, modified intention to treat, per protocol analyses]); 3) NI margin characteristics (NI margin justification [based on FDA guidelines, clinical judgment, patients’ values], NI margin selected by authors); and 4) results of the primary outcome of the study (effect size measure of primary outcome, trial conclusion [non-inferior, inferior, superior, inconclusive or no efficacy claim] based on trial results and author’s stated NI margin). We classified journals according to their impact factor as listed in Journal Citation Reports^®^ 2014 and 2019 edition.

### Risk of Bias Analysis

We evaluated risk of bias for 4 study design criteria: 1) concealment, 2) blinding, 3) analysis method, and 4) missing outcome data. Randomization was an inclusion criterion for all trials in our study, therefore, we examined risk of bias specifically due to the randomization concealment process for the second listed risk of bias criteria. Each risk of bias criteria was classified into 4 categories (low risk, probably low risk, probably high risk, and high risk) based on established methods for ascertainment of blinding and concealment.^7^ Similar categorization methods were used for analysis method, and missing outcome data. For analysis method, risk of bias was categorized based on the type of analysis used by the trial investigators as follows:

low risk (both intention to treat (ITT) analysis and per protocol (PP) analysis (gold standard analysis), probably low risk (both modified ITT and per protocol analysis), probably high risk (trials that did not state their analysis methods), high risk (used ITT, PP, or mITT analysis alone). Risk of bias for missing outcomes data was categorized according to the absolute proportion of missing outcome data and the imbalance of missing outcome data between intervention and control groups, respectively as follows: low risk (≤ 5% missing data or ≤ 2% imbalance), probably low risk (> 5 to ≤ 10% missing data or > 2% and ≤ 5% imbalance), probably high risk (> 10 to < 20% missing data or > 5 to < 10% imbalance), and high risk (≥ 20% missing data or ≥ 10% imbalance) between groups.

### Statistical Analysis

Descriptive characteristics of the trials were summarized using means, standard deviations, and medians, interquartile ratios for continuous data and proportions for categorical data. The quality of study design and risk of bias were described overall in the four categories as proportions. We also determined the proportion of trials that were categorized as low or high risk in all of the risk of bias criteria, and those that were categorized with a mix of risk of bias criteria. The risk of bias criteria in the trials were also characterized according to the subgroup of high (> = 10) and low (< 10) impact factor journals, categories that have been reported in other studies.^6^

Comparisons in proportions between groups were conducted using Chi-square, for means, using unpaired t-tests, and Mood’s test for medians. Chi square analysis was conducted to compare risk of bias according to category of journal impact factor. This study was reviewed by the Institutional Review Board at Western University of Health Sciences and received a waiver of approval.

## RESULTS

### Selection of Trials

**Figure 1:**
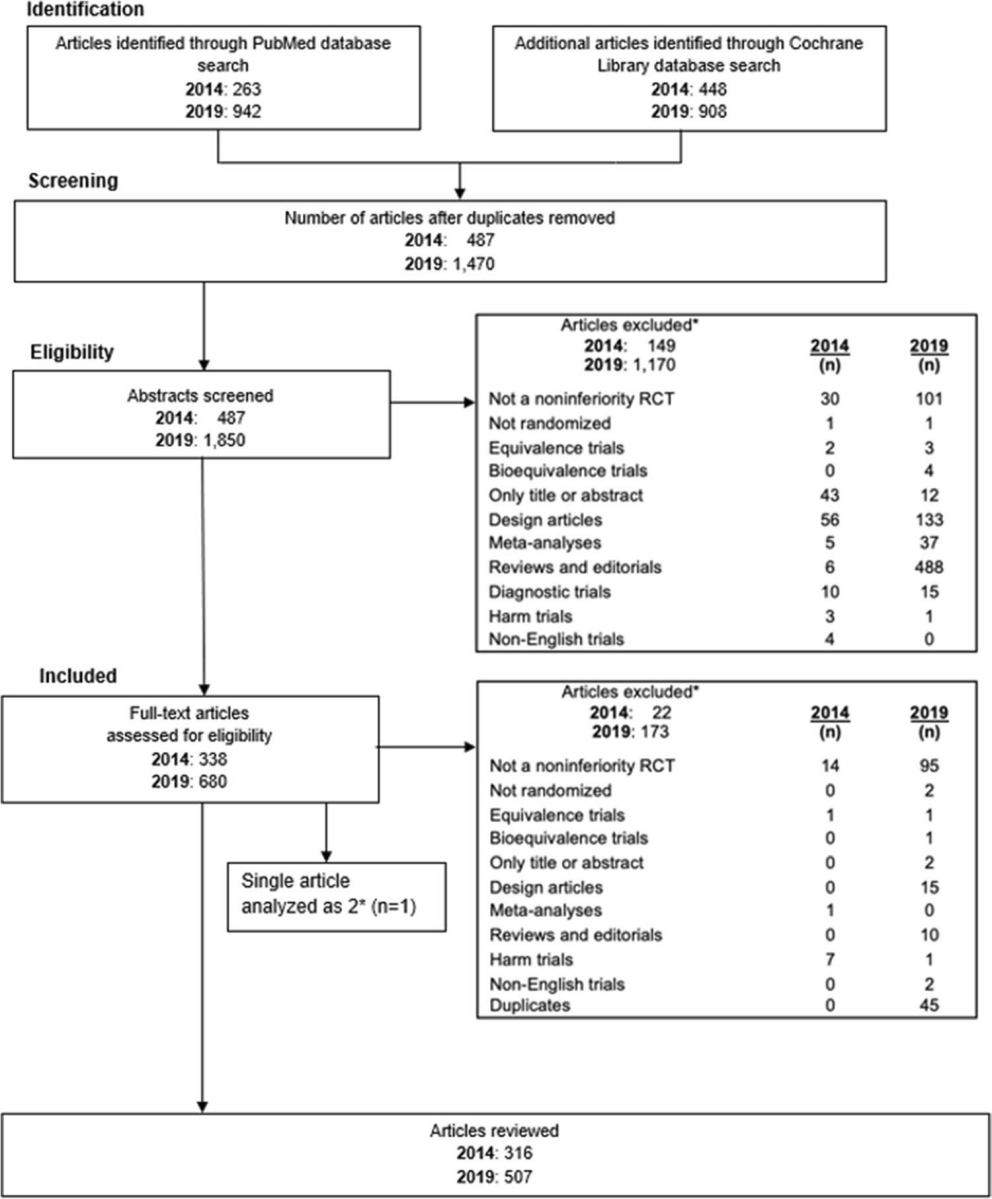
Noninferiority studies published in 2014 & 2019 and screened for inclusion and exclusion.

In 2014, the literature search yielded 487 citations. After screening the titles and abstracts, 149 studies were excluded. Upon review of the full-text articles, a further 22 studies were excluded, leaving 316 NI trials for inclusion. In 2019, the literature search yielded 1,850 citations. After screening the titles and abstracts, 1,170 articles were excluded. Upon review of the full-text articles, a further 173 articles were excluded, leaving 507 articles for inclusion. Collectively, 823 articles were included. The most frequent reasons for exclusion at the abstract/title screening phase were that the publication was a review, editorial, or design article, while in the full-text article screening phase, the trial was not a NI trial. (Fig. 1).

### Characteristics of Trials

Most trials in 2014 (n = 268, 84.8%) were published in specialty journals. While this trend remained in 2019 (n = 366, 72.2%), more trials were published in general journals (15.2% vs 27.8%, p < 0.001). No significant change was observed for the disease state focus of trials between both years, with infectious disease (21.5% vs 20.9%, p = 0.834), oncology (11.7 vs 9.1%, p = 0.261), gastroenterology (9.8% vs 7.1%, p = 0.167) and cardiology (9.5% vs 8.7%, p = 0.691) comprising the most frequent disease states. The median journal impact factor was higher in 2019 relative to 2014 (4.9 vs 4.0, p = 0.023), indicating that trials published in these journals were cited more frequently compared with those published in 2014. Most trials were designed solely as NI trials for both years, with an increase in the publication of such trials in 2019 (76.3% vs 83.4%, p = 0.026). Of these trials, a shift from investigating pharmacological to non-pharmacological interventions was seen over time (24.7% vs 37.5%, p = 0.025), with the most frequent non-pharmacological interventions including medical devices (9.9%), interventional and surgical procedures (9.3%), and care programs (5.1%). Reporting of the benefits of the new therapy to justify the NI trial design improved between years (80.7% vs 93.1%, p < 0.001), which was justified based on the intervention having improved safety compared with standard treatment (62.3% vs 68.6%, p = 0.058) (Table 1).

**Table 1:** Characteristics of Included Trials

	2014	2019	p-value
	Total, n = 316	Total, n = 507	
	Number (%)	Number (%)	
General journal	48 (15.2)	141 (27.8)	< 0.001
Journal impact factor, mean (SD)	10.4 (14.3)	15.3 (21.5)	0.001
median (IQR)	4.0 (2.6, 10.4)	4.9 (2.9, 16.0)	0.023
Total study sample size, mean (SD)	578 (853)	713.4 (2648.6)	0.424
median (IQR)	300 (120, 642)	260.5 (115, 655)	0.044
Trial design as stated by trial			0.026
Noninferiority	241 (76.3)	423 (83.4)	
Sequential: Noninferiority to superiority	74 (23.4)	81 (16.0)	
Sequential: Superiority to noninferiority	1 (0.3)	3 (0.6)	
Disease state focus of trial			
Infectious Disease	68 (21.5)	106 (20.9)	0.834
Oncology	37 (11.7)	46 (9.1)	0.261
Gastroenterology	31 (9.8)	36 (7.1)	0.167
Cardiology	30 (9.5)	44 (8.7)	0.691
Pain	27 (8.5)	20 (3.9)	0.006
Endocrinology	26 (8.2)	30 (5.9)	0.200
Genitourinary	22 (7.0)	14 (2.8)	0.004
Respiratory	19 (6.0)	25 (4.9)	0.502
Obstetrics and gynecology	17 (5.4)	23 (4.5)	0.584
Bone and joint	15 (4.7)	36 (7.1)	0.173
Ophthalmology	11 (3.5)	19 (3.7)	0.843
Psychology	10 (3.2)	12 (2.4)	0.490
Dermatology	9 (2.8)	14 (2.8)	0.941
Neurology	9 (2.8)	13 (2.6)	0.806
Nutrition	4 (1.3)	3 (0.6)	0.306
Other	17 (5.4)	25 (4.9)	0.776
Type of therapies			
Pharmacological	247 (75.0)	316 (62.3)	0.157
Anti-infective agents	50 (15.8)	63 (12.4)	0.169
Central nervous system agents	33 (10.4)	16 (3.2)	< 0.001
Hormones and synthetic substitutes	31 (9.8)	29 (5.7)	0.028
Antineoplastic agents	26 (8.2)	16 (3.2)	0.001
Serums, toxoids, and vaccines	22 (7.0)	38 (7.5)	0.775
Blood formation, coagulation, and thrombosis	15 (4.7)	24 (4.7)	0.993
Gastrointestinal drugs	13 (4.1)	6 (1.2)	0.006
Eye, ear, nose, and throat preparations	11 (3.5)	11 (2.2)	0.257
Cardiovascular drugs	9 (2.8)	8 (1.6)	0.213
Vitamins or herbal supplements	8 (2.5)	4 (0.8)	0.043
Respiratory tract agents	6 (1.9)	6 (1.2)	0.405
Immunosuppressive agents2	5 (1.6)	18 (3.6)	0.096
Bone resorption inhibitors	4 (1.3)	1 (0.2)	0.055
Disease-modifying antirheumatic drugs	4 (1.3)	10 (2.0)	0.446
Other	10 (3.2)	66 (13.0)	< 0.001
Non-pharmacological	78 (24.7)	191 (37.5)	0.025
Interventional procedures	27 (8.5)	47 (9.3)	0.723
Surgical procedures	18 (5.7)	47 (9.3)	0.064
Care programs	11 (3.5)	26 (5.1)	0.267
Medical devices	11 (3.5)	50 (9.9)	0.001
Counseling	9 (2.8)	17 (3.4)	0.687
Lifestyle modifications	2 (0.6)	4 (0.8)	0.798
Benefit of new therapy to justify non-inferiority design as noted in the publication			
Better safety profile	197 (62.3)	348 (68.6)	0.058
Improved convenience	125 (39.6)	140 (27.6)	< 0.001
Improved compliance	69 (21.8)	49 (9.7)	< 0.001
Decreased cost	49 (15.5)	109 (21.5)	0.033
Less treatment resistance	12 (3.8)	36 (7.1)	0.048
No justification provided	61 (19.3)	35 (6.9)	< 0.001

^*^SD = standard deviation, IQR = interquartile ratio

### Risk of Bias in Trials

**Figure 2:**
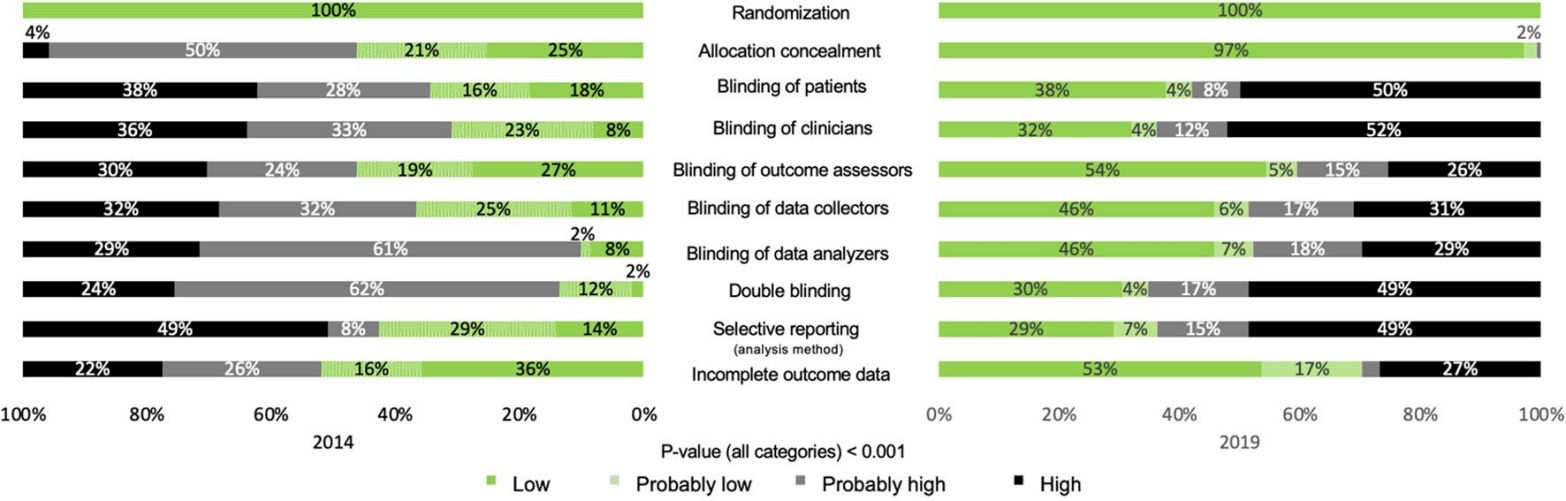
Proportion of trials with risk of bias category according to each criteria.

All trials were randomized as this was an inclusion criterion for entry into our study. Treatment allocation was concealed or probably concealed in 46.2% vs 97.2% (p < 0.001) of the trials in 2014 and 2019, respectively. There was also a significant improvement in blinding across all groups over time, with outcomes assessors being the group that was or probably was blinded the most often (46.2% vs 54.2%, p < 0.001). Only 13.6% of studies were categorized as double-blinded or as probably double-blinded in 2014 versus 36.7% in 2019 (p < 0.001). The recommended analysis method of both PP plus ITT or PP plus mITT decreased from 42.7% to 32.0%, (p < 0.001), representing fewer than half of the trials having low or probably low risk of bias. Furthermore, 8.2% vs 28.8% (p < 0.001) of trials did not specify the analysis method. Risk of bias for missing outcome data was high or probably high in 48.1% vs 29.7% (p < 0.001) in 2014 and 2019, respectively (Fig. 2). For NI trials published in 2014, 25.0% had a high journal impact factor (≥ 10) compared with 27.2% in 2019. No difference was observed for the four risk of bias design criteria between low vs. high journal impact factor within the 2014 and 2019 years, and no changes were found over time. (Appendix Figs. 1–4).

### Description of Non-inferiority Margin in Trials

The NI margin was specified in nearly all trials for both years (94.3% vs 95.1%), with an increase in the number of trials determining the NI margin pre-randomization (88.3% vs 94.5%, p = 0.001). Reporting of the rationale for how the size of the margin was determined increased over time (36.7% vs 56.8%, p < 0.001). While clinical judgement remained the most common source (29.8% vs 22.5%), there was an increase in the number of articles incorporating patient values as justification for the NI margin (0.3% vs 20.7%, p < 0.001). The source of the expected active control event rate was described in about half of the trials for both years (55.1% vs 47.3%, p < 0.001), and was mostly based on single or multiple trials. (Table 2) Notably, there was an increase in articles presenting primary outcomes as absolute (41.8% vs 47.9%; p < 0.001) rather than relative values, with a mean NI margin of 11 vs 10 (p = 0.308) for absolute values and a mean ratio difference of 1.36 vs 1.13 (p = 0.350) for relative values over time. Overall, there was a slight but significant change in the main conclusions for NI trials published in 2014 and 2019, respectively: 77.5%/79.1% (non-inferiority), 2.8%/3.8% (inferiority), 7.0%/2.8% (superiority), and 12.7%/14.4% (inconclusive, including not non-inferior, not inferior and not superior) (p = 0.032).

**Table 2:** Quality of Reporting Methodology for Noninferiority Margin

	2014	2019	p-value
	Total, n = 316	Total, n = 507	
	Number (%)	Number (%)	
Noninferiority margin reported	298 (94.3)	482 (95.1)	0.631
NI margin determined prior to randomization	279 (88.3)	479 (94.5)	0.001
Control Event Rate/Efficacy Source			< 0.001
Opinion of study group	62 (19.6)	35 (6.9)	
Singular trial	33 (10.4)	99 (19.5)	
Multiple trials	28 (8.9)	75 (14.8)	
Analysis performed by study group	13 (4.1)	54 (10.7)	
SR or meta-analysis	6 (1.9)	4 (0.8)	< 0.001
Not stated	174 (55.1)	240 (47.3)	< 0.001
Authors report amount of efficacy retained by NI margin	10 (3.2)	26 (5.1)	0.180
Justification for NI margin	116 (36.7)	288 (56.8)	< 0.001
Clinical judgment	94 (29.8)	114 (22.5)	0.020
FDA recommendations Patient Values	21 (6.6) 1 (0.3)	69 (13.6) 105 (20.7)	0.002 < 0.001
Not stated	200 (63.3)	219 (43.2)	0.000
Noninferiority margin not reported	18 (5.7)	26 (5.1)	0.631
Primary endpoint reported as:			< 0.001
Absolute risk difference	132 (41.8)	243 (47.9)	
Relative risk	2 (0.6)	32 (6.3)	
Odds ratio	1 (0.3)	13 (2.6)	
Hazard ratio	16 (5.1)	50 (9.9)	
Other (mean score, length of stay, etc.)	165 (52.2)	161 (31.8)	
Margin as stated (mean, SD)			
Absolute risk difference	11 (6.4)	10 (6.9)	0.308
Ratio difference (HR, OR, RR)	1.36 (0.23)	1.13 (1.05)	0.350

^*^SR = systematic review, SD = standard deviation, HR = hazard ratio, OR = odds ratio, RR = relative risk

## DISCUSSION

The findings of our systematic review demonstrate that the methodological quality and reporting of NI trials, although improved over time, continue to be suboptimal as more than half of the studies evaluated were designed as open-label studies, did not utilize the gold-standard method of analysis, and lacked justification for the NI margin, along with other deficiencies. With medical practice steered by evidence-based literature, the increasing publication of NI trials in general journals with higher impact factors demonstrates the frequency that such studies are encountered in clinical practice, and the potential impact they can have in clinical decision-making.

The CONSORT statement recommends blinding in NI trial design, as it may influence the validity of such trials. We found that while most studies evaluated were designed as open-label studies, there was an improvement from prior studies and between both years. Prior studies that evaluated methodological quality and reporting of NI trials either did not evaluate the use of blinding or demonstrated that less than half of the NI trials evaluated utilized blinding, with the majority of such trials encompassing an open-label design.^6,8–10^ Investigators and clinicians must be aware that open-label design biases towards non-inferiority due to potential for cointervention and contamination, and lack of a placebo effect.

Moreover, per CONSORT, both ITT and PP analysis are recommended to assess whether conclusions are stable across analyses in NI trials in estimating the treatment effect. Our study demonstrated suboptimal method of analysis for NI trials published in both years, as only one-third utilized the “gold-standard” of ITT and PP for analysis method, with approximately one-fourth of the remaining studies in 2014 and 2019 potentially biased towards a conclusion of non-inferiority by using ITT analysis alone. Furthermore, there was a concerning increase in the number of NI trials that failed to report the specific analysis method used to evaluate the primary outcome studied. These findings did not change based on journal impact factor, as there was no difference in the risk of bias design criteria between low vs high journal impact factor trials.

Prior studies have demonstrated significant variability in the proportion of NI trials that utilized the “gold standard” of ITT and PP analysis, ranging from 10–70%, exemplifying deficiencies in reporting the method of analysis.^4,5,8,10–14^ In addition to reporting only one method of analysis, such findings may be attributed to how different analysis methods such ITT, PP, and mITT are defined within individual NI trials. This ambiguity can influence inferences made based on how these methods of analyses are defined and reported.

While missing outcome data in any trial may be inevitable, its impact may compromise the integrity and interpretability of NI trials even more because of reduced statistical power, increased Type 2 error rates and the potential of biased estimates of the treatment effect size.

Prior studies demonstrated that up to 42% of NI trials had missing outcome data.^4,8^ While our study demonstrated improved handling of missing outcome data from prior studies and between both years, up to one third of NI trials published in 2019 were found to have high or probably high risk of bias related to missing outcome data. Per CONSORT, it is imperative for trials to clearly report if imputation methods are used to handle missing data. Additionally, trials should specify what method is used as well as any assumptions made to minimize biases that may threaten the validity, and more importantly, the conclusions of such trials.

Notably, the primary outcome of the trials in our study changed only slightly between both years, with approximately three-fourths of the studies evaluated concluding NI. Prior studies have demonstrated variable rates of concluding NI, ranging from 48–90%, which may be attributed to such studies being limited to certain specialties or investigating specific interventions.^4,5,8–11,13,14^ Of note, similar studies have demonstrated that despite the majority of NI trials concluding NI, up to one third of such trials reported potentially misleading conclusions, including superiority or inferiority, due to a discordance between the results presented and the conclusions provided by NI trial investigators, based on FDA recommendations.^5,8,11^ Thus, journals that publish NI trials must examine for inadequate trial methods that can result in such limitations and potential risks of bias, and ensure they are noted for the audience, regardless of the journal impact factor.

When designing and conducting NI trials, it is imperative to provide justification for the NI trial design, which is often based on the novel therapy offering a benefit or advantage over the standard of care such as improved safety, lower cost or greater convenience. Our study demonstrated improved reporting of the benefit of the new therapy to justify a NI trial design among trials published in 2019 compared to those published in 2014, which was justified based on the intervention having improved safety compared with standard treatment. Similar studies either did not evaluate this or reported the benefit of the new therapy in a generalized manner, such as “improved patient outcomes.”^8,10^ Explicit justification for the NI trial design is warranted to provide clinicians with an understanding of what advantages the novel therapy may offer to account for the proposed loss of efficacy in improving patient outcomes.

The NI margin is an essential component to the design and conduct of NI trials, as it is the reference used for concluding NI. Per CONSORT, it is recommended that this margin is chosen based on clinical judgement. In our study, the NI margin was specified in nearly all trials published in both years, with an increase in the number of trials determining the NI margin prior to randomization. Our findings remain relatively unchanged to prior studies, which found that over 90% of the NI trials evaluated reported the NI margin.^4,5,8,9,12^

While most articles reported a NI margin, only half provided justification for the size of the NI margin, which was predominantly based on clinical judgement with increased consideration of patient values. Additionally, the source of the expected active control event rate was described in about half of the trials, primarily based on single/multiple trials. Although justification of the NI margin was low, this was an improvement from previous studies which demonstrated that 30–45% of NI trials provided justification for the NI margin, which was predominately based on clinical judgment or historical data.^4,5,8,9,12^ Principally, a previous study demonstrated that NI margins vary widely and frequently exceed absolute treatment benefits on outcomes seen in cardiovascular superiority trials, emphasizing the need for investigators to determine and prespecify the benefits and harms of the novel treatment when determining the NI margin.^15^ Lack of justification for the NI margin can lead to too much loss of efficacy, which could lead to utilization of inferior drugs within clinical practice. Thus, transparency in the methods used to determine the NI margin can aid clinicians in determining whether the NI margin and the rationale for the margin’s choice influenced the validity of the results.

While the results of our study demonstrate that there continues to be deficiencies in the methodological quality and reporting of NI trials per CONSORT recommendations, it is important to recognize that the NI trial design is inherently susceptible to bias despite adherence to CONSORT recommendations, which may impact the accuracy and interpretability of the results. The bias depends on how investigators define non-inferiority, including the NI margin. Claims of non-inferiority based on the author-defined NI margin may not accurately reflect the true efficacy of the novel therapy compared with the standard of care.^16^ For example, if a NI study findings are statistically significantly in favor of the standard of care, the new therapy may not be declared inferior, and may instead be represented as non-inferior or inconclusive. Such conclusions can have profound impacts on clinical practice, as they can potentially lead to implementation of inferior therapies in clinical practice.

To the best of our knowledge, this is the largest and most comprehensive study that examined the methodological quality and reporting of NI trials. A strength of our systematic review is that it was conducted without restriction to journal type, journal impact factor, disease state or intervention and compared the quality of these trials over time. Additionally, our study investigated if published NI trials reported the benefit of the new therapy to justify the NI trial design. Prior studies evaluating the methodological quality and reporting of NI trials either did not evaluate this or reported the benefit of the new therapy in a manner that was determined to be too broad to derive meaningful inferences. Moreover, while prior studies evaluated specific risk of biases related to NI trials, including blinding and method of analysis, our study performed a comprehensive risk of bias assessment of published NI trials including randomization, concealment, blinding, method of analysis and missing outcome data.

Our systematic review has some limitations. First, the NI trials evaluated were limited to those published in English, therefore, results may differ for published non-English NI trials. The search terms, filters, and the number of search databases used may not have captured all NI trials eligible for inclusion. However, our search strategy was quite comprehensive, and since we excluded many trials in the abstract and full-text reviews, our search terms were likely broad enough. Third, we did not search for any gray literature. Lastly, we used 2019 as the most recent comparison year, which was 5 years after 2014. These two time periods were chosen in our study, as they allowed for sufficient time for published NI trials to incorporate the 2010 CONSORT recommendations and detect any improvements over time. Additionally, while 2019 is not the most recent year of data available, due to the COVID-19 pandemic, it was our belief that trials published in 2020 may have differed in quality and may not have accurately reflected trends in the quality of reporting of NI trials.

## CONCLUSION

The findings of our systematic review demonstrate that although the methods and quality of reporting of NI trials are improving, there continues to be deficiencies in the design and conduct of NI trials, specifically related to justification of the NI margin, blinding, and method of analysis. Clear justification of the NI margin is essential for accurate interpretation of NI trial results, and minimizes the potential for an unacceptable loss of efficacy with subsequent introduction of inferior therapies into clinical practice. These deficiencies may be attributed to unique aspects of NI trials compared with superiority trials, creating unfamiliarity of the investigators, authors, reviewers and editors of journals. Thus, it remains imperative that researchers, institutional review boards, editors and peer reviewers develop greater awareness of and strive for improved compliance with available guidelines that serve to improve the quality of reporting and reliability of results and conclusions of NI studies. Improved descriptions of the methods used and justification for the design and analysis of such trials is warranted to ensure accurate and consistent delivery of information that could hold vital implications in clinical decision-making.

## Supplementary Information

Below is the link to the electronic supplementary material.

Supplementary file1 (DOCX 70 KB)
