# Supplementary material for: Non-Inferiority Trials: A Systematic Review on Methodological Quality and Reporting Standards
Source: J Gen Intern Med. 2024 Jul 1;39(13):2522–30. doi: 10.1007/s11606-024-08890-9 (PMC11436551; doi:10.1007/s11606-024-08890-9)
Supplement: Supplementary file 1 — Supplementary file1 (DOCX 70 KB) [file 11606_2024_8890_MOESM1_ESM.docx]

**SUPLEMENTARY INFORMATION**

**Appendix**

**Appendix Figure 1. (2014) Risk of Bias Summary of Non-inferiority Trials in Journals with a Low (<10) Impact Factor (n = 237, 75.0%)**


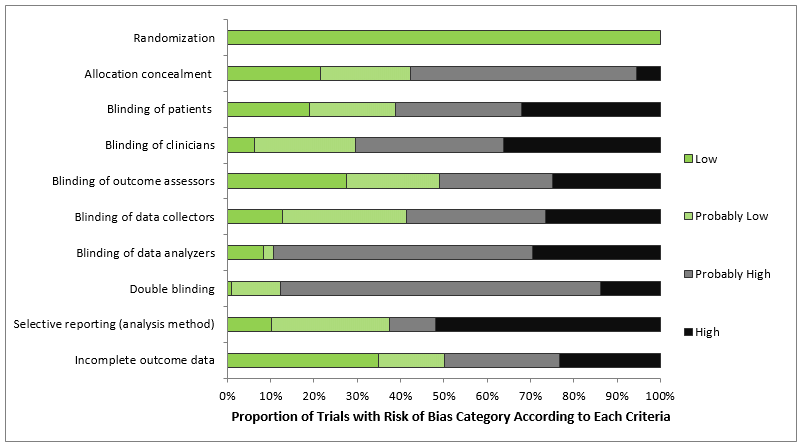


**Appendix Figure 2. (2014) Risk of Bias Summary of Non-inferiority Trials in Journals with a High (>10) Impact Factor (n = 79, 25.0%)**


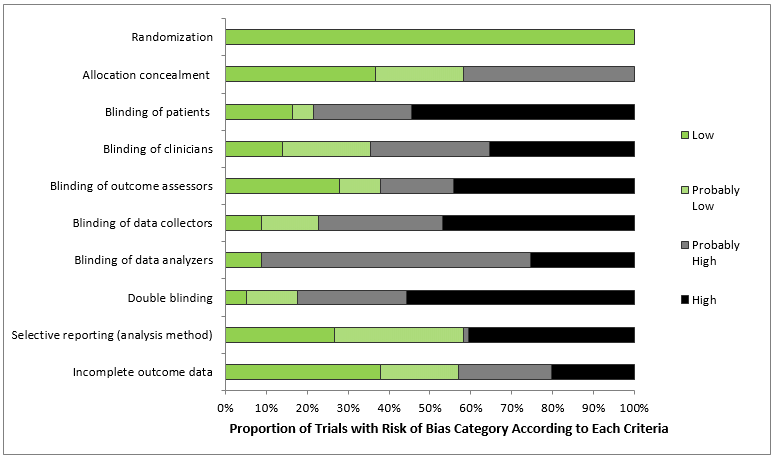


**Appendix Figure 3. (2019) Risk of Bias Summary of Non-inferiority Trials in Journals with a Low (<10) Impact Factor (n = 337, 66.4%)**

**Appendix Figure 4. (2019) Risk of Bias Summary of Non-inferiority Trials in Journals with a High (>10) Impact Factor (n = 138, 27.2%)**
